# Indexing Strategies for Rapid Searches of Short Words in Genome Sequences

**DOI:** 10.1371/journal.pone.0000579

**Published:** 2007-06-27

**Authors:** Christian Iseli, Giovanna Ambrosini, Philipp Bucher, C. Victor Jongeneel

**Affiliations:** 1 Ludwig Institute for Cancer Research, Bâtiment Génopode, Université de Lausanne, Lausanne, Switzerland; 2 Swiss Institute of Bioinformatics, Bâtiment Génopode, Université de Lausanne, Lausanne, Switzerland; 3 Swiss Institute for Experimental Cancer Research, Epalinges, Switzerland; National Cancer Institute at Frederick, United States of America

## Abstract

Searching for matches between large collections of short (14–30 nucleotides) words and sequence databases comprising full genomes or transcriptomes is a common task in biological sequence analysis. We investigated the performance of simple indexing strategies for handling such tasks and developed two programs, fetchGWI and tagger, that index either the database or the query set. Either strategy outperforms megablast for searches with more than 10,000 probes. FetchGWI is shown to be a versatile tool for rapidly searching multiple genomes, whose performance is limited in most cases by the speed of access to the filesystem. We have made publicly available a Web interface for searching the human, mouse, and several other genomes and transcriptomes with oligonucleotide queries.

## Introduction

One of the challenges of the post-genomic era is to be able to identify rapidly, in fully sequenced genomes, transcriptomes, and variants thereof, exact or near-exact matches to short words with a limited number of occurrences. Such words could be the sequences of individual probes from Affymetrix[1] or other gene chips, PCR primers for the amplification of unique genomic regions (STS[2]), tags derived from SAGE[3], [4] or MPSS[5], [6] experiments, or the raw data from next-generation high-throughput sequencers (using e.g. the Illumina technology). Applications include probe and PCR primer design, mapping of probe sets to genome features, interpretation of digital gene expression analysis experiments, or individual genome re-sequencing. Ideally, software for word matching should be able to accomplish the following tasks: (i) search very large databases (≫10^9^ nucleotides) efficiently; (ii) return accurate and fully exhaustive results for short (14–30 nucleotides) query sequences; (iii) scale up gracefully to large collections of queries (10^6^ queries); (iv) provide a mechanism for finding single- or multiple-nucleotide mismatches in alignments that are global relative to the query.

To our knowledge, no software has yet been designed to precisely match these specifications. Megablast[7], a variant of the NCBI BLAST suite that uses a word index to reduce the database search space, and then a greedy algorithm to align only highly conserved regions, can be tweaked to efficiently find exact matches to short sequences (see below), although this was not part of its original design. SSAHA[8], which creates a database hash table in memory to accelerate search functions and was thus expected to perform reasonably well, is optimized to find longer alignments and fails in practice on most of the criteria set out above. The indexing strategy used by the PCR primer analysis program of Jim Kent (http://genome.ucsc.edu/cgi-bin/hgPcr?command = start, http://www.soe.ucsc.edu/∼kent/src/) is probably the approach that comes closest to our goals, but it is meant to work with primer pairs and not with independent queries.

For fast performance, the implementation of SSAHA or BLAT keep the index or the hash table in the memory. They only use non overlapping words and may use repeat masked sequence. Thus for many of the short sequence searches they may not find any or all matches. In contrast, the fetchGWI and tagger programs use a complete indexing strategy for every base in the genome or other databases and thus have higher sensitivity in sacrifice of speed. This is desirable for certain applications.

In order to screen single or multiple genomes with collections of sequence tags of various sizes, we designed two programs with complementary goals and approaches, fetchGWI and tagger. Tagger builds an index in RAM from a set of query sequences, and is particularly well-suited for generating one-time mappings of large collections of tags (typically all probes on a standard Affymetrix chip set or a Nimblegen tiling array, or all tags derived from a SAGE experiment) onto a genome or transcriptome. FetchGWI relies on pre-computed genome indices stored on disk, and is best used in cases where a limited number of queries have to be mapped very rapidly, e.g. as a component of a Web service. Only fetchGWI will be described in detail here, as it performs as well or better than tagger in all tests, provided precomputed genome or transcriptome indices are available.

For the occasional user, fetchGWI may be the fastest connection from a small piece of a gene sequence to a genome browser window displaying information about the genomic environment of the corresponding gene. To this end, we developed a web interface that provides direct HTML links to Ensembl[9] UCSC[10], and NCBI[11] genome browsers for each exact or near-exact matches to a query target found in a collection of large sequence databases. Sequence databases comprise entire genomes or mRNA reference sequences[12] (i.e. human, mouse, drosophila, dog, rat and chimpanzee). This web service can be used interactively, or via sequence tag-associated hyperlinks from within text documents.

## Methods

### Design Considerations

Genome sequences are high-quality, so we need to consider only the four unambiguous nucleotides A, C, G, and T. The information necessary to represent those 4 nucleotides can be encoded on 2 bits of data. Current processors are built to easily handle 64-bit words, so it is straightforward to encode up to 32 nucleotides within a single data word.

To get maximal search speed, we want to search only within the index file. Thus we need one index entry for each nucleotide in the genome. This exhaustive index also ensures that no match can possibly be missed, as can happen when only non overlapping words are used to construct the index.

To further improve search speed within the index file, we also implemented a second format, which we call a compressed index file, where the primary index file is subdivided into 16,777,216 (2^24^) parts according to the first 12 nucleotides of each word. The compressed format also allows a 25% size reduction of the index file.

The method is divided into two separate steps: generation of the index files, and actual sequence search. The generation of index files needs to be performed once for each new version of a genome or transcriptome. The search step can be repeated as often as necessary once the index files have been generated.

### Index Files Structure

The main index file is composed of a sorted list of entries. Each entry is composed of four parts: a sequence tag, two flags, a sequence offset, and an accession number index.

#### sequence tag

**Figure 1:**
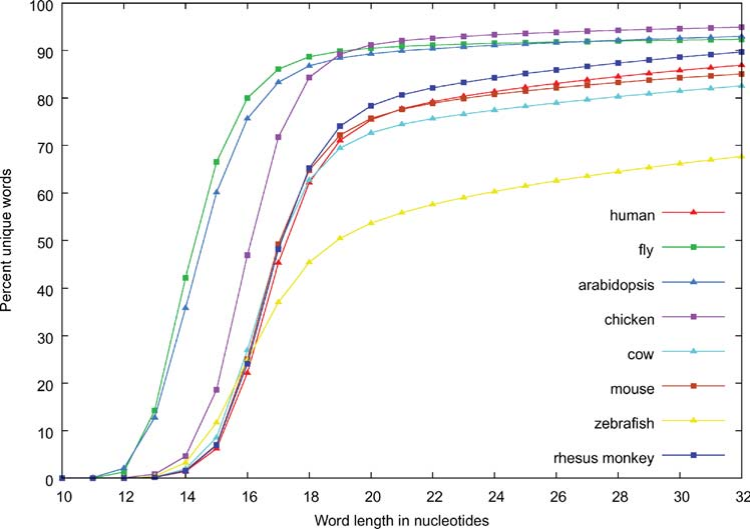
Word duplication in several genomes. Analysis of the percentage of unique sequence tags in several genomes as a function of tag length.

The first and largest part is the sequence tag, which is a binary encoding of a piece of DNA sequence. Each nucleotide is encoded using two bits. The optimal size of the sequence tag is a compromise between two opposing goals: while longer sequence tags are more specific, and less likely to occur at multiple positions in the genome, shorter sequence tags make for smaller index files and shorter search times. We analyzed the percentage of unique sequence tags, in a number of complete genomes, as a function of tag length (Fig. 1). The data show that all curves have an inflection point located between 15 and 20 nucleotides. For example, in the human genome most tags shorter than 16 nucleotides are not unique as expected from the fact that the size of the human genome is comprised between 4^15^ and 4^16^. We also took into account the fact that many current methodologies (SAGE, MPSS, Affymetrix or Nimblegen genome arrays, PCR primers, etc.) use tags where the size ranges in length from 17 to 25 nucleotides. We thus decided to use a tag size of 25 nucleotides or 50 bits of sequence.

#### flags

The second component of an index entry is two flags: one marking incomplete sequence tags, the other selecting short or long sequence offset size.

While each sequence tag is 25 nucleotides long, we would still like to allow shorter queries, with possible matches at the ends of database sequences. This causes a problem near gaps and sequence ends, because sub-words within the last 25 nucleotides would not be indexed unless we allow for the indexing of incomplete (i.e., shorter than 25) sequence tags. In this context, a gap refers to a single or consecutive run of ambiguous nucleotide characters, usually N's. Note that in the current genome sequence data files, short gaps of unsequenced regions are represented by runs of 50–100 N's. The flag is here to mark such shortened index entries. The smallest query allowed is 10 nucleotides long. To be more specific, such a word will be padded by a run of complemented nucleotides of the last valid nucleotide to make it a valid 25-letter word, and the incomplete flag will be set. For example, the 10-mer ATGGCTGAGG will be stored as ATGGCTGAGGCCCCCCCCCCCCCCC, the flag will be set, and it is easy to find back the original word by removing all occurrences of the repeated last nucleotide.

We have analyzed the genomic and mRNA content of the RefSeq database, release 22. The longest sequence is 748,055,161 nucleotides long (chromosome 1 from the gray short-tailed opossum), so at least 30 bits are needed to specify an offset within such a large sequence. However, most sequences are shorter. There are 1,769,766 sequences in the analyzed release, and only 5,330 are longer than 1,048,576 nucleotides. To help decrease the size of the index, we decided to use a flag to distinguish between sequences which need large offset values but are few in numbers, and sequences which can use a shorter offset but are more numerous.

#### sequence offset

The third index component is the offset of the sequence tag within the sequence. As noted in the previous paragraph, the longest sequence currently known is roughly 750 megabases long so we need 30 bits to encode the long offsets. The short offset uses 20 bits and can thus be used for sequences up to 1,048,576 nucleotides long.

#### accession number index

The last component is used to retrieve the accession number (or other unique identifier) of the sequence from which the sequence tag was derived. A list of all the accession numbers of the indexed sequences is kept in an auxiliary index file. This auxiliary file is a plain text file, where each sequence accession number is given a unique index number, sequentially, starting from 0 for long sequences and from 16,384 for the shorter ones. We have set aside 14 bits to encode the index number for long sequences, and 24 bits for shorter sequences. We can thus accommodate 16,384 long sequences and 16,760,832 short sequences, which is currently more than enough for all genomes and transcriptomes found in RefSeq and other (e.g. FANTOM, H-INV) collections.

The complete index entry uses 96 bits, or 12 bytes, of storage. Given that the human genome is roughly 3 billion nucleotides long, the index file for this genome will occupy about 30 gigabytes of storage space. With today's hard disks storing 500 gigabytes apiece, this is a manageable size.

### Compressed Index Files

The idea behind the compressed index is to diminish the size of the index files and to improve filesystem locality when searching multiple words. Filesystem locality can improve the search speed by reducing the amount of time spent seeking between different parts of the hard disks and by taking advantage of data caching. The whole index file is logically subdivided into 16,777,216 parts, each part representing words having identical first 12 nucleotides. A secondary index, recording the position in the main index file where each part starts, is added to the end of the index file.

As an added benefit, since the first 12 nucleotides of each word are given through the secondary index, the first 3 bytes of each entry in the main index can be suppressed thus saving 25% of storage space. The space required to store the secondary index is 128 megabytes, which is negligible.

### Index File Generation

The generation of an index file is performed in two steps:

1. the genwin program is used to transform a set of FASTA formatted files, containing nucleotide sequences, into a set of files containing unsorted index entries, in the format explained in the previous paragraphs. The generated files are not sorted nor compressed. They must be sorted using the sortGWI program before being usable by fetchGWI. Short words down to 10 nucleotides long are indexed, using the incomplete flag explained above
2. the sortGWI program is used to sort and/or merge the files generated in step 1 into a file containing sorted index entries; the sortGWI program can generate and use both plain and compressed index files

### Searching Through the Index Files

Finding where a query matches in the genomic sequence is accomplished through the fetchGWI program. This program performs the following steps:

1. collect all the queries to be searched, either from command line arguments or from text files containing one query per line. Each query line must contain a sequence tag to be searched at the beginning of the line, optionally followed by a non-alphabetical character and arbitrary data on the rest of the line, which will be copied verbatim in the output. The sequence tag can contain degenerate nucleotides (the standard letters BDHKMNRSVWY are accepted), which will be automatically expanded into all possible matching tags
2. if the user specified a search with one or several mismatches, generate all possible sequence tags to be searched by replacing the specified number of non-degenerate nucleotides with all other possibilities
3. add all the reverse complemented queries, unless the user specified otherwise, so that the search is performed on both strands
4. split queries longer than 25 nucleotides into 25 nucleotide sub-queries, keeping proper linking with the original queries. Queries shorter than 25 nucleotides are padded on the right with the nucleotide A to 25 nucleotides and a mask is generated to allow proper comparison with the index by masking out the unused nucleotides on the end of both the query and the index
5. sort all the queries using the same sorting order as the index files, so that we can benefit from better filesystem locality when performing the search
6. map the secondary index structure in memory (when using compressed index files)
7. perform the search of each query within the index file, using a dichotomic search (also known as binary search) technique[13] and collect all the matches for each query. When using compressed index files, a lookup in the secondary index is performed to determine the boundaries of the dichotomy search within the main index. The index is masked to the proper length during each comparison for queries shorter than 25 nucleotides
8. for queries longer than 25 nucleotides, analyze the matches of the sub-queries and keep only those compatible with the original query. This is performed by sorting all the matches by queries, and examining all the sub-queries to find those occurring in the proper arrangement
9. report the results by appending the actual sequence tag found, along with the accession number and position offset within the sequence for each matched query lines

It is possible to search short queries down to 10 nucleotides long exhaustively, since short words down to 10 nucleotides are indexed. It is possible to force fetchGWI to seek also shorter queries, but in this case some words can be missed near gaps and sequence ends.

### The tagger Program

There are cases where constructing the index file itself is considered too time consuming, usually because it would be used only once. For these cases, we also supply a program named tagger which uses a similar indexing strategy, but in the case of tagger the index is built from the queries instead of the genome, and is only kept in RAM. The word size is 13 nucleotides and the index table uses 512 megabytes of memory. Each tag to be searched is entered in the table, and they are kept in a linked list when several tags are attached to the same index location in the table. This is a very simple hashing technique. The tagger program then parses through FASTA formatted files containing the sequences to search and reports matches as it finds them. All searched tags must be the same length and cannot contain ambiguous nucleotides, which are further restrictions compared with the fetchGWI program.

### Tested programs

To test and validate our approach, seeking only exact matches for the queries, we compared the output results and execution speed of our fetchGWI and tagger tools with the results obtained by megablast.

It should be noted that searches using megablast produced two different results:

1. the query TTGTGTTGTGTTGTGTTGTGTTGTG found only 58 matches whereas 94 are expected on the current assembly of the human genome. This is probably because megablast reports non-overlapping matches, which is a matter of choice – note that the above sequence is a repeat of the 5-letter word GTGTT
2. the query TGAATTCGGTCTTGCCTTGAACACA found a spurious perfect match on the genomic sequence NGAATTCGGTCTTGCCTTGAACACA.

Except for these minor problems, all three tools reported the same hits. The version of megablast was 2.2.13. The parameters used for the megablast runs were the following:

-f T -J F -F F -W 12 -s 25 -D 0

We also attempted to perform the same searches using SSAHA (Version 3.2), but it only would match 24 of the 25 nucleotides of the query sequences (the reason for this behavior is unknown to the authors).

We then proceeded to test results when seeking possibly inexact matches for the queries. However, neither megablast, nor blast, were able to retrieve all correct hits reliably. For example, the sequence ATGGCTGAAGGCCTTATGAGTCAAA has one exact match on human chromosome 12 (NC_000012.10[10029321..10029345]) and 5 inexact matches (2 mismatched nucleotides: ATGGCTGAGGGCCTTAAGAGTCAAA) on human chromosomes 5, 10, 11, X, and Y. FetchGWI finds all matches in about 10 seconds Megablast only finds the exact match and 2 of the 5 inexact ones in roughly 6 minutes, while blast finds the exact match only (in about 10 seconds). The parameters used for megablast in this case were:

-f T -J F -F F -W 8 -s 21 -D 0 -q -1 -G 10 -E 4

**Figure 2:**
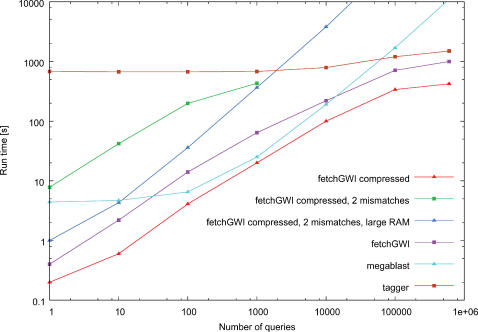
Runtime comparisons on the human genome. Runtime comparisons between fetchGWI using plain and compressed index files, tagger, and megablast. Each point is computed from the average of three runs on the human genome with different input data, except the last run done on the whole dataset. Only perfect matches are sought, except for the 2 experiments explicitly noted were 2 mismatches were allowed.

**Figure 3:**
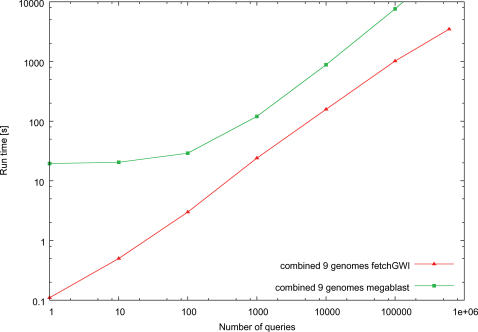
Runtime comparisons on multiple genomes. Runtime comparisons between fetchGWI (using a compressed index file) and megablast. Each point is computed from the average of three runs on the combined genome of 9 species (human, mouse, honey bee, cattle, dog, drosophila, zebrafish, chimp, and rat) with different input data, except the last run done on the whole dataset. Only perfect matches were sought.

**Figure 4:**
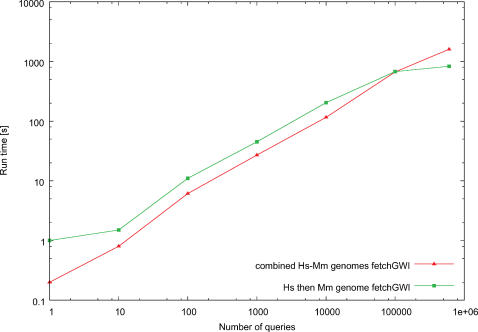
Runtime comparisons on combined index files. Runtime comparisons of fetchGWI when using either multiple index files, or a single, combined, index file. Each point is computed from the average of three runs on the human and mouse genomes with different input data, except the last run done on the whole dataset Only perfect matches were sought.

**Figure 5:**
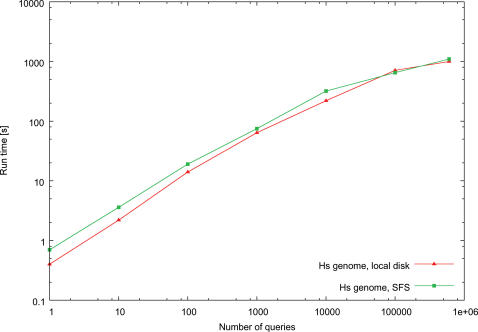
Runtime comparisons on different filesystems. Runtime comparisons of fetchGWI when the index file is stored either on a filesystem on local disks, or on a SFS cluster filesystem. Each point is computed from the average of three runs with different input data, except the last run done on the whole dataset. Only perfect matches were sought.

The fetchGWI and tagger results of Fig. 2, Fig. 3, and Fig. 4 were computed on a Linux workstation with Intel Xeon processors running at 3 GHz, 4 GB RAM, and a local SATA hard disk of 250 GB. The megablast results were computed on a Linux workstation with Intel Xeon processors running at 3.4 GHz in em64t mode, 8 GB RAM, and local hard disks of 128 GB in striped RAID mode. The fetchGWI results on the SFS (a commercial implementation of LUSTRE from Cluster file systems, inc., http://www.lustre.org/.) filesystem (Fig. 5) were computed on a Linux workstation with Intel Itanium2 processors running at 1.3 GHz, 4 GB RAM, and an 8 TB SFS filesystem attached through an InfiniBand network.

## Results and Discussion

To examine the behavior of the fetchGWI and the tagger programs, we ran queries with variable numbers of tag sequences on the human and mouse genomes. Only exact matches were sought, except for two experiments reported in Fig. 2. For building the queries, we used the probes from the Affymetrix “Human Genome U133 Plus 2.0” array. The reason for choosing this probe set is that it should offer a pretty good coverage of the whole human genome, and it contains a reasonably sized set of 604,258 tags.

The test queries were generated in the following way:

- take the first three probes, and create three files with a single tag
- take the next thirty probes, and create three files of ten tags
- continue the procedure up to three files of one hundred thousand tags
- the whole 604,258 tags file is also used as the final test

Each query set (three files each containing 1 to 100,000 tags, and one with 604,258 tags) was searched against assembly 36 of the human genome, using fetchGWI, tagger, and megablast.

The average wall clock run time for the searches is plotted in Fig. 2. The CPU time needed by fetchGWI is only a few percent of the wall clock time; most of the time needed is spent waiting for data to be retrieved from the filesystem. The CPU time needed by both tagger and megablast is nearly the same as the total wall clock time; most of the time needed is spent doing computations. As expected, the search time of tagger is essentially independent of the number of queries; however, it is somewhat surprising that tagger never outperformed fetchGWI, even for very large query sets. The use of compressed index speeds up the search 3- to 4-fold for small number of tags, and is still slightly faster for large number of tags. It is also worth noting that for query sets with 1000 tags, megablast performs almost as well as fetchGWI, provided the correct command-line switches are used. However, fetchGWI is over 10-fold faster than megablast for mapping all 604,258 probes of the Hu133 chip; larger query sets such as those found on genome-wide tiling arrays are expected to show an even larger advantage for fetchGWI. The speed advantage of fetchGWI is even slightly better when searching through several genomes at once, as shown in Fig. 3.

We also show in Fig. 2 the run time of fetchGWI when allowing 2 mismatches, and note that it increases by a factor between 10 and 100. The search space is much larger, since it will grow as the binomial coefficient of n and k, where n is the number of nucleotides in the query and k is the number of mismatches allowed. For example, searching for one query of 25 nucleotides and allowing 2 mismatches is equivalent to searching for 2,776 queries with no mismatches. During the tests we performed, the maximum amount of RAM used when searching for 1000 queries and allowing 2 mismatches was 550 megabytes on a 32-bit machine and 700 megabytes on a 64-bit machine.

The run time of a dichotomic search is *O*(ln*n*), where *n* is the number of elements in the database. When the number of queries gets large, some additional time will be spent sorting the queries themselves, where the standard Unix quicksort routine is used and has a run time of *O*(*n* · ln*n*) where *n* is the number of queries. What we observe in the results is the large influence of filesystem data caching by the operating system. For example, repeating a search containing 1000 queries brings the wall clock time down from around 90 seconds to 0.15 second. On a machine with sufficient amounts of RAM, even very large searches can be performed in less than 1 second once the data have been cached. When the set of queries gets large, the same data caching effect starts to show (flattening of the curve with query sets of more than 10,000). The reason is that fetchGWI sorts the queries, and thus the denser the hits get on the genome, the higher the chance that the next query to be searched corresponds to index data already loaded in RAM.

One of our goals was to provide a quick means to search for matches across all known genomes, and this raises the question of whether to keep each genome in its own index file, or to produce index files of combined genomes. The behavior of fetchGWI has been plotted in Fig. 4 for the following two cases:

1. produce a combined index file for the human and mouse genomes, which has a size of 50 gigabytes and takes around 10 minutes to produce from the individual index files of the human and mouse genomes
2. search first through the human genome index file, then repeat the search for the mouse genome

Figure 4 shows that the performance gain for combining the two indices is minimal. Again the influence of caching is apparent: for large query sets, the difference disappears. For small query sets, the *O*(ln*n*) behavior of the dichotomic search is the key.

Dealing with very large files is not an easy task, and it makes sense to use computer clusters to speed things up further. We therefore analyzed the behavior of fetchGWI on a server that gets its data from a distributed SFS cluster filesystem, to see how such a system would cope with multiple random accesses distributed through large files. Figure 5 shows that the results are very similar to those measured for local hard disks. This indicates that multiple instances of fetchGWI should be able to run efficiently on a modern compute cluster with LUSTRE-based file sharing.

### Availability and requirements

We have created a web portal, TagScan at http://www.isrec.isb-sib.ch/tagger, for rapid mapping of short oligo sequences to large sequence databases comprising full genomes or transcriptomes (i.e. full length mRNA sequences from human, mouse, bee, drosophila, dog, rat, cow, and chimpanzee). The web server implementation responds to the need of having a page that lets the user screen vertebrate genomes or transcriptomes with oligo sequences (on average 19–25 nucleotides long) and returns both perfect matches and one or two nucleotide mismatches. This could certainly be a very useful service for people designing PCR primers or oligos for custom arrays. Other potential uses could include tasks such as remapping large number of probe sequences from SNP or tiling arrays to new assemblies of the human or other genomes. The TagScan web server supports searches for perfect matches and for single or double nucleotide mismatches. Along with word matches, genomic or transcript coordinates are returned. For genome-wide searches, useful hyperlinks to popular genome browsers such as the Ensembl, UCSC and NCBI ones, are also provided.

Alternatively, the TagScan programs can be invoked directly by using tag-associated hyperlinks from within text documents. The hyperlink format is shown below:

1. http://www.isrec.isb-sib.ch/cgi-bin/tagger/tagscan?dbtype = dna&dbname = HS&mode = 0&tag = ATGAGGTATTAGGAT

Further details on the use of inline hyperlinks are available from the web page.

The TagScan inline URL service provides an easy and elegant mechanism for specialized databases to hyper-link sequence-tagged features to genome browsers without explicitly providing chromosomal coordinates. The advantage of this on-the-fly mapping mechanism is that it doesn't require recalculation of genome coordinates for new assemblies by the client database.

The source code is freely available on the SourceForge server:

- Project name: Tagger
- Project home page: http://sourceforge.net/projects/tagger
- Operating system(s): All POSIX
- Programming language: C
- Other requirements: none
- License: GNU GPL

The distribution also contains a user manual in the form of man pages for the provided programs. There is a description of the index structures and access functions for prospective developers in the source code itself.

### Conclusions and Perspectives

Tagger and fetchGWI were originally developed to support research and development programs at our parent institutions. The first application was to find sequences proximal to EST-derived polyadenylation sites in the human genome[14]. Tagger was subsequently used to remap the eukaryotic promoters in EPD[15] to new assemblies of the human and other genomes with the aid of unique sequence tags of length 60, and to reliably associate “historically annotated” Affymetrix probe sets with newly annotated genes and transcripts[16]. It was also essential in producing a reliable annotation for human and mouse MPSS signatures[6].

The programs were designed for speed and robustness rather than for elegance or flexibility. They have been extensively field-tested, and shown to produce accurate results very rapidly. The results shown here indicate that their main strength lies in the matching of very large probe collections to one or more genomes. As such, they may become the sequence similarity search engines of choice for developers of complex arrays of probes while keeping track of issues of within-species and cross-species hybridization. We also found fetchGWI extremely useful for tracking the source(s) of contaminants in biological samples.

We have for the first time presented benchmark results to assess the efficiencies of different indexing strategies for rapid exact sequence matching in realistic settings. Our results indicate that a compressed sorted word-index accessed by dichotomic search outperforms other approaches for mapping large collections of short tags to large genomes by an exact match criterion.

The fetchGWI and tagger programs could serve as search engines for a large variety of other applications. We are currently developing a tool using a modified version of fetchGWI for rapid location of weight matrix-defined transcription factor binding sites in whole genomes. The underlying principle is to expand the user supplied weight matrix to the complete set of k-words that match the matrix with scores equal to or higher than a threshold value. A virtually infinite number of heuristic sequence similarity search algorithms could use a fetchGWI-like mechanism as a first filtering step. The index structures and search algorithms described here are straightforward to use by application programmers. The benchmarks presented will enable interested developers to judge whether a fetchGWI type index structure could provide an efficient solution to a specific problem arising in a whole genome or transcriptome scan application.
